# Immunopeptides: immunomodulatory strategies and prospects for ocular immunity applications

**DOI:** 10.3389/fimmu.2024.1406762

**Published:** 2024-07-15

**Authors:** Yi Tang, Sheng Qu, Zichao Ning, Hong Wu

**Affiliations:** Eye Center of Second Hospital of Jilin University, Changchun, China

**Keywords:** immunopeptides, autoimmune disease, inflammatory, ocular diseases, immunity

## Abstract

Immunopeptides have low toxicity, low immunogenicity and targeting, and broad application prospects in drug delivery and assembly, which are diverse in application strategies and drug combinations. Immunopeptides are particularly important for regulating ocular immune homeostasis, as the eye is an immune-privileged organ. Immunopeptides have advantages in adaptive immunity and innate immunity, treating eye immune-related diseases by regulating T cells, B cells, immune checkpoints, and cytokines. This article summarizes the application strategies of immunopeptides in innate immunity and adaptive immunity, including autoimmunity, infection, vaccine strategies, and tumors. Furthermore, it focuses on the mechanisms of immunopeptides in mediating ocular immunity (autoimmune diseases, inflammatory storms, and tumors). Moreover, it reviews immunopeptides’ application strategies and the therapeutic potential of immunopeptides in the eye. We expect the immune peptide to get attention in treating eye diseases and to provide a direction for eye disease immune peptide research.

## 1 Introduction

The immune imbalance of the eye brings great trouble to treating eye diseases (such as dry eye, uveitis, microbial infections, and tumors). As the blood-retina barrier (BRB) causes the immune privileges of the ocular, ocular surface (1, 2), the central nervous system, and retina (3, 4) secrete peptides and cytokines like TGF-β2 to regulate resident cells, resist pathogen invasion, and maintain ocular immune microenvironment (5–7). Since peptides maintain the immune microenvironment with diverse immune effects, understanding the pathogenesis of immune peptides in eye diseases is essential for developing effective treatments.

Immunological peptides are biologically active molecules composed of amino acids and amino acid residues, which are diverse in immunomodulatory capabilities. In a literature review, Robert E.W. Hancock et al. summarized the immune regulatory function of host defense peptides (HDPs), which includes harmonizing pro-inflammatory and anti-inflammatory reflections and chemoattraction, enhancing bacterial killing and cellular differentiation, activating immune compartments, wound healing, and autophagy, as well as apoptosis and pyroptosis (8). Initially, HDPs and antimicrobial peptides (AMPs) were primarily used in the antimicrobial field. Subsequently, some HDPs and AMPs were observed to regulate cytokines and immune systems (9–12). Recently, immunopeptides have been active in cancer, autoimmune disease, and inflammation research. The development and application of peptide drugs have been closely watched in the past decades, with more than 600 kinds of polypeptide drugs in clinical and preclinical trials (13). By 2020, more than 100 kinds of peptides will be approved for the treatment or diagnosis of peptides (14). Introducing immune peptides into eye disease treatment is promising due to the eye’s immune-privileged status and its ability to produce immune antigens and HDPs for ocular immune regulation.

**Figure 1:**
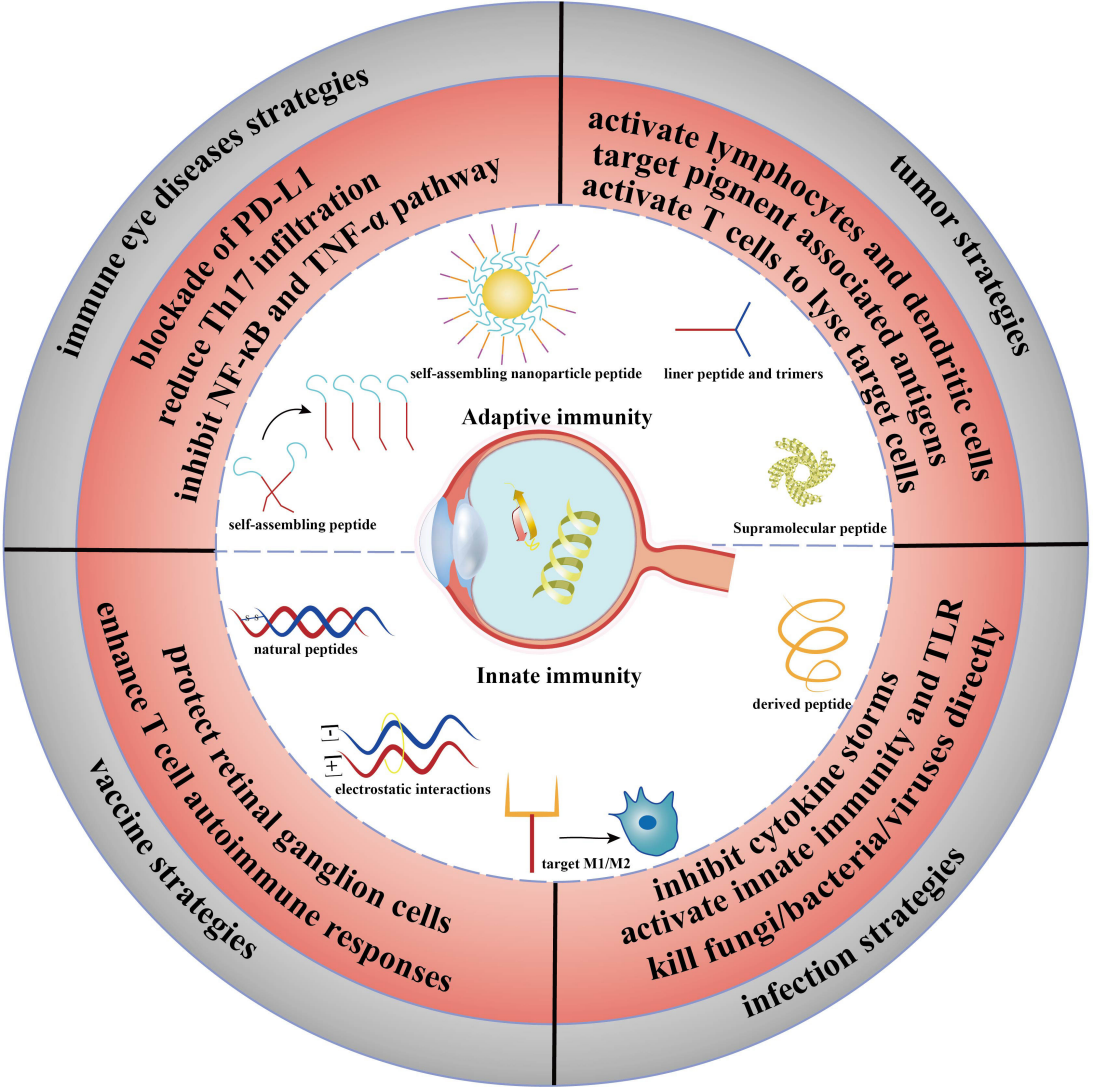
Abstract figure of the application of immunopeptides in ophthalmic diseases. The immune strategy of peptides in adaptive immunity and innate immunity. Immune eye diseases strategies: DED, uveitis; vaccine strategies: glaucoma; infection strategies: keratitis and endophthalmitis; tumor strategies: UMS.

Here, we reviewed the mechanisms of immunological peptides in mediating innate immunity and adaptive immunity and explored the application potential of immune peptides. Furthermore, we analyzed the application progress of immunological peptides in ocular immune diseases and infectious diseases, intending to identify a new direction for immunopeptides in eye diseases (**Figure 1**).

## 2 Mechanism of immunological peptides

### 2.1 Regulating innate immunity

Innate immunity primarily defends against ‘invaders’ or pathogens rapidly, inducing acute inflammation (15). Macrophages and dendritic cells (DCs) are the primary immune cells, and pattern recognition receptors (PRRs) are responsible for perceiving pathogen-associated molecular patterns (PAMPs) and damage-associated molecular patterns (DAMPs) (16). PRRs contain TLRs, NLRs, and C-type lectin receptors, among others (16). Especially, TLRs are vital to mediate inflammation and exogenous defense. TLR contains three functional domains (17) and forms a cytoplasmic juxtamembrane α-helix (18): i) leucine-rich repeats (LRRs); ii) a transmembrane domain; iii) a Toll interleukin (IL)-1 receptor (TIR) domain. The domains help to identify viral and bacterial components to activate the downstream pathways. Subsequently, TLRs trigger the Myeloid differentiation primary response-88 (MyD88)-NF-κB pathway or TIR-domain-containing adapter-inducing interferon-β (TRIF), stimulating inflammatory factors like TNF-α, the interleukins family (such as IL-1β, IL-4), TGF-β, and chemokines (16, 17). NLRs are proteins with a nucleotide-binding domain (NBD/NOD) at the N terminal and a leucine-rich repeat (LRR) domain at the C-terminal, which enables them to recognize a wide range of ligands (19, 20). NOD1 recognizes diaminopimelic acid (gamma-d-glu-meso-Diaminopimelic acid (iE-DAP)) on the cell wall of Gram-negative bacteria (G-), while NOD2 recognizes muramyl dipeptides and viral ssRNA. The immune processes of NOD2 activation (20, 21). is that the activated macrophages engulfed pathogens to form phagosomes, which fuse with lysosomes to form phagolysosomes. Furthermore, the lysosomes decompose the cell walls into peptidoglycan, producing muramyldi peptides. Then muramyldi peptides enter the cytoplasm to activate NOD2. Activated NOD2 and serine-threonine protein 2 (RIP2) form dimers and recruit caspase activation and recruitment domain (CARD), leading to downstream activation of TAK1 and NF-κB (21).

Immunological peptides regulate innate immunity by binding to LPS or interacting with PAMPs (**Table 1**). When innate immunity is activated, epithelial, inflammatory, and immune cells produce HDPs to promote or inhibit inflammation. Immune peptides can also play an antibacterial role and regulate the body’s immunity. This chapter details the mechanism of different HDPs regulating innate immunity.

**Table 1:** Peptides in innate immunity.

Name	Sequence	Source	Innate immunity	Refences
LL-37	LLGDFFRKSKEKIGKEFKRIVQRIKDFLRNLVPRTES	Neutrophils, Monocytes, Mast cells, Epithelial cells	Recruit neutrophil. Upregulating pro-inflammatory factors and cytokines. Overexpression activates autoimmunity.	(22–24)
Human α-defensins	X_1-2_CxCRx_2-3_Cx;ExgGxCx_3_Gx_5_CCX_1-4_	Neutrophils, Paneth cells	Mediate macrophage to secrete PMN.	(25)
Human β-defensins	X_2-10_Cx_5-6_ (G/A)xCX_3-4_Cx_9-13_Cx_4-7_CCx	Neutrophils, Epithelial	Stimulate IL-6, IL-10, IFN-γ. Upregulate the secretion of IP-10 and MCP -1. HBD1 might inhibit EGFR-VEGF pathway.	(26, 27) (28)
Bac2A	RLARIVVIRVAR-NH2	Bactenecin	Induce chemotaxis of undifferentiated THP-1 cells.	(29)
IDR-1018	VRLIVAVRIWRR-NH_2_	Bac2A	Resist Candida albicans infection. Upregulate MCP-1 and IL-10. Inhibit the expression of IL-1β, IL-6 and IL-12.	(30)
IDR-1002	VQRWLIVWRIRK-NH2	Bac2A	Promote chemokines. Recruit neutrophils and mononuclear/macrophages. Inhibit IFN-γ and IFN-regulatory factor-8 regulated networks.	(31) (32) (33)
Clavanin-MO	FLPIIVFQFLGKIIHHVGNFVHGFSHVF-NH2	Hemocytes of marine tunicates	Upregulate IL-10, GM-CSF, IFN-γ, MCP-1. Downregulate IL-12 and TNF-α. Chemotaxis.	(34)
M4	WQR-NH2	Litopenaeus vannamei heads	Promote the release of NO. Strengthen macrophages to devour Upregulate TNF-α, IL-2, IL-6 and IL-1β.	(35)
ToAP3	FIGMIPGLIGGLISAIK-NH2	Tityus obscurus	Reduce pro-inflammatory factors TNF-α and IL-1β.	(36)
ToAP4	FFSLIPSLIGGLVSAIK-NH2	Tityus obscurus	Reduce pro-inflammatory factors TNF-α and IL-1β. Up-regulates IL-10. T-cell-mediated immune regulation.	(36)
M2pep	YEQDPWGVKWWY	Derived synthetic peptide	Target type2 macrophage.	(37)
RP-182	KFRKAFKRFF	Derived synthetic peptide	Trigger CD206 on M2 to induce endocytosis and apoptosis. Enhancing the innate immune.	(38)
Histatin-1	DpSHEKRHHGYRRKFHEKHHSHREFPFYGDYGSNYLYDN	Mucosal tissue	Inhibited macrophage releasing proinflammatory cytokines. Inhibit MAPK signaling pathways. Reduce NO release to protect ocular surface tissues.	(39)
IDR1	KSRIVPAIPVSLL-NH2	Derived synthetic peptide	Immunomodulatory peptides with chemotactic activity on neutrophils, monocytes, and macrophages. IDR1 plays an immunomodulatory role and reduces the expression of pro-inflammatory factors through MAPK and other pathways.	(12)

The mammalian cathelicidin (LL-37) is HDP that works in inflammatory injuries and tumor microenvironments (40). LL-37 plays different immune roles in different environments. To adjust inflammation, LL-37 recruits neutrophils to inflammatory sites (22) and promotes the differentiation of monocytes into immature DCs, which in turn upregulates pro-inflammatory factors (CXCL1, CCL2, and CCL7) and cytokines (IL-6 and IL-8) (41, 42). LL-37 can down-regulate the signal transduction of TLR4 to play an anti-inflammatory role, act as a bridge between innate immunity and adaptive immunity, and upregulate the production of Th1 cell polarization cytokines (43). However, in severe infections, LL-37 inhibits neutrophil infiltration and migration through focal adhesion kinase (FAK), extracellular signal-regulated kinase (ERK), and p38 pathways (41). It also induces neutrophil apoptosis by increasing the levels of the antiapoptotic protein Bcl-XL. In the tumor microenvironment, LL-37 is out of control and dysregulated. Overexpressed LL-37 inhibits immune function and promotes the differentiation of macrophages to M2, promoting tumorigenesis. On the other hand, LL-37 also enhances the anti-tumor effects of CpG oligodeoxynucleotides by stimulating immunocompetent cells, leading to increased expression of IFN-γ and natural killer (NK) cells (44). In autoimmune diseases, overexpressed LL-37 leads to overactivation of TLR9, causing an over-enhanced adaptive response (23). In psoriasis, LL-37 binds to its DNA to form an immune complex and binds to TLR9 to release IFN-α from plasma cell DCs (45).

Human α-defensins and β-defensins are another family of defensins with a potent pro-inflammatory function. Human α-defensins (Human neutrophil peptides 1–3, HNP1-3) showed chemotactic effects on monocytes, immature DC, and naive CD4+ T cells (46). HNP1-3 mediates macrophage to secrete PMN, which triggers the powerful release of Fcγ receptors CD32 and CD64 (25). In addition, HNP1 promotes the release of TNF-α from PBMCs and may also destroy pro-IL-1β (47). Human β-defensins (HBD) are important in autoimmunity as the dysregulation of defensin expression would lead to autoinflammatory and autoimmune diseases. HBD3 might be an autophagy activator in atopic dermatitis (48). HBD3 stimulates PBMCs via TLR-MyD88-NF-κB pathway and IRAK-1 (49). It also improves the expression of CD86, CD80, and CD40 on DCs and monocytes (49). The activated NLRP3 inflammasomes trigger exogenous HBD to inhibit IL-1β secretion (50). HBD1 is released by keratinocytes, a potential chromosome 8p tumor suppressor that might inhibit the EGFR-VEGF pathway (26, 28). And it upregulates costimulatory molecules CD80, CD86, and CD40, induces TNF-α, IL-6, and IL-12P70, and enhances DC-mediated T-cell proliferation (51). HBD2-4 stimulates IL-6, IL-10, and IFN-γ, enhancing the secretion of IP-10 and MCP -1 (27).

**Figure 2:**
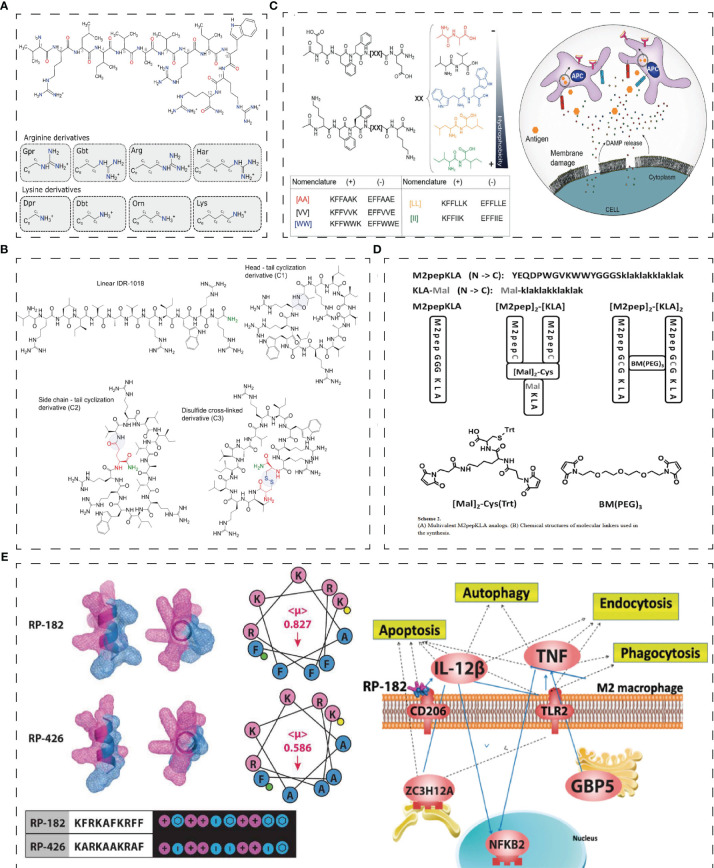
Structure of innate immunity peptides. **(A)** Skeletal diagrams of IDR-1018 and side chains of the cationic amino acid derivatives (52). **(B)** Chemical structures of the IDR-1018 and cyclic derivatives (53). **(C)** PAIIRpeptide based on the coOP strategy (promote electrostatic interactions between two oppositely charged aas) (54). **(D)** Multivalent M2pepKLA analogs and chemical structures of molecular linkers used in the synthesis (55). **(E)** Secondary-helical structures of RP-182 and RP-426 and pathway studio graph of GO Cell Processes of the most common genes across enriched gene sets in RP-182 treated M2 BMDMs (38). Reprinted with permission from journal/author license.

Bac2A, a 12-amino acid linear derivative of bactenecin, is chemotactic to undifferentiated THP-1 cells (29). Hancock et al. (31) designed a series of synthetic innate defense regulatory peptides (IDR) based on bovine lysopeptide Bac2A, among which IDR-1 and IDR-1002 can regulate innate immunity to resist bacterial invasion by inducing chemokines. IDR-1002 encourages the generation of chemokines and recruits immune cells like neutrophils and mononuclear/macrophages to the infection site to enhance protection against bacterial infection (32). The attachment of neutrophils was boosted in a manner dependent on integrin-2 (31). IDR-1002 also controls inflammatory signaling by inhibiting IFN-γ and IFN-regulatory factor-8-regulated networks (33). IDR-1018, another derivative of Bac2A, can also resist the bacteremia of Candida albicans infection, upregulate MCP-1 and IL-10 levels in mice, and inhibit the expression of IL-1β, IL-6, and IL-12 (30). In recent years, researchers have found that adding non-natural amino acids to HDPs can regulate the immune activity of HDPs. At the same time, replacing cationic or hydrophobic residues with non-natural amino acids can enhance the stability of proteolysis. Hancock et al. (52) utilized non-natural cationic amino acids with differing lengths of side chains in the synthetic HDP to synthesize the innate defense regulatory peptide (IDR) through the SPOT polypeptide array (**Figure 2A**). The derivatives of IDR-1018 show up-regulation or down-regulation of IL-1β and MCP-1, which is related to non-natural cationic amino acids. Hancock et al. (53) found that the immunoregulatory function of IDR-1018 cyclic peptide (**Figure 2B**) was more sensitive to IL-1R regulation after treatment with head-tail cyclization, glutamate side-chain to tail cyclization, and disulfide bond cyclization.

Muramyl peptides are a component of bacterial peptidoglycan, broken down by lytic enzymes and trigger immunity. Muramyl peptides have been studied extensively in clinical trials. Muramyl peptides activate immune cells (like DCs, macrophages, T lymphocytes, B lymphocytes, neutrophils, and NK cells), mediating the inflammatory process and immune tolerance. Meanwhile, muramyl peptide is activated by NAKG phosphorylation (56), interacting with NLRs and triggering a signaling cascade of reactions (57), which induces IL-1, IL-2, IL-6, IL-8, IL-12, TNF-α, IFN, MHC, and CSF. Furthermore, muramyl peptides respond to TLR4 and TLR2 agonists, activating the NOD2 receptor and inducing the cascade of NF-κB-MAPK and the IRF pathway (58).

M1-M4 are immunological peptides prepared from Litopenaeus vannamei heads (35). Studies proved that M4 could bind to TLR2/TLR4-MD2, significantly promote macrophages to product NO, enhance the phagocytic ability, and remarkably increase the release of TNF-α, IL-1β, IL-6, and IL-6 (35). The Hong Kong oyster mainly relies on innate immunity to resist microbial invasion. chGnRH, a neuroimmune peptide, increased in expression after Vibrio infection in oysters from Hong Kong (59). In addition, GnRH promoted the expression of inflammatory mediators and cytophagocytosis through cAMP and its downstream signaling pathway protein kinase APKA (59). Clavanin-MO (34), derived from marine tunicate peptides, stimulates leukocytes at the infection site and produces GM-CSF, IFN-γ, and MCP-1. At the same time, Clavanin-MO could downregulate IL-12 and TNF-α to repress harmful inflammation (34). ToAP3 (FIGMIPGLIGGLISAIK-NH2) and ToAP4 (FFSLIPSLIGGLVSAIK-NH2), two peptides derived from Tityus obscurus, have similar immunomodulatory mechanisms to Clavanin-MO that ToAP3 and ToAP4 had immunomodulatory effects on LPS-treated BMDM cells, and the pro-inflammatory factors TNF-α and IL-1β were reduced at both molecular and transcriptional levels. In addition, ToAP4 up-regulates IL-10 levels and is involved in T-cell-mediated immune regulation (36).

In addition to regulating inflammation, peptides can be used as vaccines to regulate innate immunity. Developing peptide-based vaccines can help reduce the adverse immune reactions associated with vaccines and improve immune efficiency. Kang Zhou et al. (60) designed HM3 (IRRADRAAVPGGGGRGD-NH2), an angiogenesis inhibitor peptide with an Arg-Gly-Asp sequence, which can target tumor cells and inhibit tumor growth (61). Furthermore, FITC-HM3, as a switch, mediates cognate tumor cells releasing lower levels of cytokines (62). However, a limitation of vaccine peptides is low immunogenicity (63). Adding TLR agonists and adjuvants to immune peptides can effectively enhance the body’s innate immune response. Mitchell, R. A. et al. showed that co-delivery of CS peptide, TLR7/8 and TLR9, agonists and adjuvant AddaVax through the skin can effectively improve the innate immune response of the body by expressing proinflammatory factors and chemokines such as IL-6, IL-12, and IP-10 (64) and reduce cell invasion.

As the first defence of innate immunity, M2-like TAM can directly promote tumor growth by releasing cancer-promoting factors, or indirectly promote tumor growth through neovascular, cancer stem cells, or immunoaggressive microenvironments. Therefore, peptides targeting macrophages are an essential direction in tumor therapy. M2-like macrophages are associated with asthma, allergic inflammation, and the pathological progression of tumors. M2pep (YEQDPWGVKWWY), an anti-tumor peptide targeting TAM, can accumulate in combination with TAM in mice, ultimately improving the survival rate of tumor-bearing mice (37). Additionally, combining the M2pep peptide with pro-apoptotic KLA peptides [M2pep]_2_- [KLA]_2_ and [M2pep]_2_-[KLA] (**Figure 2D**) enhances the cytotoxicity of M2 macrophages *in vitro* (55). Furthermore, the peptide Melittin (MEL) can bind to CD206 M2 macrophages, where its binding to the amphiphilic cationic helical peptide KLA increases mitochondrial-induced programmed cell death to achieve the effect of targeted M2 cell therapy (65). In addition, IDR RP-182 is an immunotherapy agent derived from IDR (**Figure 2E**). RP-182 can trigger CD206 on M2 to induce endocytosis and apoptosis and transform M2-like macrophages into M1, enhancing the innate immune response (38). Moreover, RP-182 can interact with PD-L1 to increase antitumor immunogenicity (38).

Gunay G. et al. (54) have designed a Peptide Aggregation Induced Immunogenic Rupture (PAIIR) peptide based on the coOP strategy (promote electrostatic interactions between two oppositely charged amino acids), which generates immunogenic cell death (ICD) and activates innate immunity (**Figure 2C**) —the gathering PAIIR would cause membrane damage, releasing DAMPs and triggering focal inflammation. Suresh Babu, V. et al. designed a series of cationic amino acid CAPs with antibacterial activity and good cell penetration as the sequence was rich in guanidian groups in arginine (66). They found that HC3 and HC5, *in vitro*, disrupted Bcl2/Bax expression to damage the mitochondria, activate the caspase cascade, and guide WERI - Rb1 cell apoptosis (66).

### 2.2 Modulating adaptive immunity

Adaptive immunity includes T-cell-mediated cellular and B-cell-mediated humoral immunity (**Table 2**). Unlike innate immunity, adaptive immunity has specificity, memory, and the distinction between self and non-self. There are four signals to activate naive T cells: 1. T cell antigen receptor recognition. 2. Antigen-presenting cells (APCs) provide co-stimulation. 3. Cytokine environment differentiates T cells into protective T cell subsets or increases the indirect effects of co-stimulatory molecules on APC (cytokines increase the effect of T cells) (77, 78). 4. MADS recognition: Metabolic RF activates immune checkpoint molecules through metabolic sensors, consecutively activating APCs and facilitating the expression of cytokines (78).

**Table 2:** Peptides in adaptive immunity.

Name	Sequence	Target cell	Adapt immunity	Refences
SNP-TLR7/8a	SIINFEKL-VFPRSPTVFYNIPPMPLPPSQL	T cell	Enhance neoantigen-specific T cell responses. Activate human DC subsets. Promote CD4 and CD8 T cell immunity.	(67)
Coil29	QARILEADAEILR-AYARILEAHAEILRAQ	T cell	Activate T cell response.	(68)
PAQ11	H2N-SSLENFRAYV-SGSG-QQKFQFQFEQQ-Am	T cell	Improved CD8+ T cell response time.	(69)
TP-AP	SC (DBCO)FPNWSLRPMNQM- MDEKAQKGPAKLVFFAC (Cy)EK (N3)G	PD-L1	Prolongation of the occupancy time of PD-L1. Promote T cell proliferation.	(70)
CLP002/ CLP003	WHRSYYTWNLNT/WHFSYNWRWLPP	PD-L1	Blocking the effect between PD-L1 and PD-1. Restore the T cell proliferation.	(13)
Human α-defensins	X_1-2_CxCRx_2-3_Cx;ExgGxCx_3_Gx_5_CCX_1-4_	T cells	Trigger Ag-specific IgG and IgM Ab. Induce CD4+ T cell proliferation and chemotactic to T cells Release IL-5, IFN-γ, IL-6 and IL-10	(26) (71)
Histatin-1	DpSHEKRHHGYRRKFHEKHHSHREFPFYGDYGSNYLYDN	Mucosal tissue	Decrease CD45 cell infiltration.	(39)
VIP	HSDAVFTDNYTRLRKQMAVKKYLNSILN	CD4+ T cell	Inhibit macrophage proinflammatory actions Promote a positive Th2/Th1 balance	(72)
α-MSH	HFRW	T cell	Inhibit the secretion of IFN-γ. Promote the transformation of T cells into Tregs. Curtail the ability of effector T cells to activate and proliferate.	(73)
R16	ADGSSWEGVGVVPDV	T cell	Inhibition of T cell activity. Reduction of optic nerve damage.	(74)
KS23	KDKAMLFTYDQYQENNVDQASGS	Th1 cell Th17 cell	Activate AMPK. Inhibit NF-κB signaling pathway to limit autoimmune inflammation. Reduced the release of TNF-α, IFN-γ, IL-17 and IL-23A. Decreased the proportion of Th1 and Th17 cells in peripheral blood.	(75)
B27PD	ALNEDLSSWTAADT	T cell	Reduce ocular inflammation by decreasing cytokines (IL - 4, IL - 10 and TGF - β). Lowered the activity of effector cells.	(76)

The major histocompatibility complex (MHC) and immune checkpoint are important targets of immunopeptide action. MHC, including MHC-I and MHC-II molecules, binds peptides during antigen presentation to activate T cells. MHC-I can bind peptides in the 8–10 amino acid range, and MHC-II class molecules bind peptides in the 12–15 amino acid residue range (77). MHC molecules bind to broken intracellular antigens and form complexes that are released onto the cell surface. These complexes are recognized by CD8+ T cells, which then clear them through cytotoxicity. On the other hand, MHC-II molecules bind to exogenous antigens, which are then degraded into amino acids within the cell. These amino acids are recognized by CD4+ T cells, which activate humoral immunity (79). Therefore, it is of great significance to analyze the binding domain of MHC molecules to peptides and use “immunopeptiomics” to build up the structure of immune peptides through mass spectrometry (79, 80). The immune checkpoint plays a vital role in T cells’ physiological function, including effector function, differentiation, and loss. Immune checkpoint ligands on APCs bind to T cell receptors, mediating the inhibition or activation of T cell function in tumors. B-cell-mediated humoral immunity functions mainly through antibodies, including T-cell-dependent and non-T-cell-dependent pathways (81). In T-cell immunity, B cells are activated by antigen recognition, immune checkpoint CD40/CD40L binding to activate B cells, and cytokines that enhance the B cell response (81).

**Figure 3:**
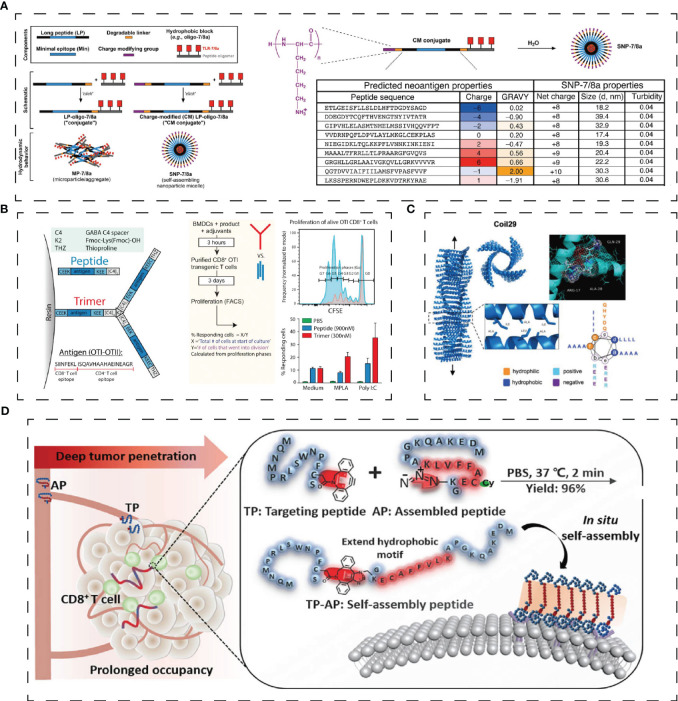
Structure of adaptive immunity peptides. **(A)** Schematic of modular components comprising peptide-TLR-7/8a conjugate vaccines and charge-modified (CM) peptide-TLR-7/8a conjugate vaccines that form microparticles/aggregates (MP-7/8a) and self-assembling nanoparticle micelles (67). **(B)** Solid phase synthesis of linear long peptides and trimers produces products that can be processed and presented by (bone marrow-derived) dendritic cells to CD8 þ T cells *in vitro* (83). **(C)** 3D structure of fiber-forming peptide Coil29 (PDB 3j89) and helical wheel projection of peptide sequence (68). **(D)** TP targets ligand programmed death-ligand 1, pointedly identifying cancer cells and then self-assembled into nanofibers TP-AP (70). Reprinted with permission from journal/author license.

T cell immune regulation can administer T cells proliferation and activities targeting different activation signals and cells. T Peptides that target T cells are mainly used in the form of vaccines. Peptide-based vaccines have minimal side effects and bind to specific HLA domains by synthesizing tumor-associated or specific peptide combinations, then binding to CD4 and CD8 T cells to activate T cell responses (63). The vaccine with melanoma peptide and tetanus adjuvant peptide can elicit a strong T-cell response when paired with an incomplete Frankenstein adjuvant and a TLR receptor agonist (82). Geoffrey M et al. (67) have developed a vaccine platform, ‘SNP-TLR7/8a’, which co-delivers peptide-based tumor antigens with molecularly defined adjuvants (**Figure 3A**). It can be systematically improved to boost the intensity and breadth of the responses of neoantigen-specific T cell and widely mobilize human DC subsets to generate crucial cytokines that promote Th1 CD4 and CD8 immunity (67). Furthermore, short MHCI-like peptides that can induce CD8+ lead to T cell dysfunction at the inoculation site, while prolonged peptides contribute to T cell activation. S. T. T. et al. (83) designed a trimer antigen MAtriX to enhance T cell immunogenicity (**Figure 3B**). The core of the design lies in the lysine at the end of the long peptide, which has alpha and e amino groups that extend out from the second and third branches in parallel. This structure is able to accommodate a variety of antigens and help inhibit the growth of tumors in the body. As synthetic long peptides (SLPs) consist of CTL and Th epitopes, they are sensitive to adaptive immunity. However, SLPs are also prone to enzymatic degradation and are quickly cleared. Wim E. Hennink et al. designed cationic dextran nanogels to load SLPs into a polymeric network, resulting in superior CD8+ T cell responses compared to soluble peptides and nanogel formulations with physically loaded peptides, both *in vitro* and *in vivo* (84).

The supramolecular peptide vaccine consists of epitope peptides that contain α-helix structures capable of self-assembling into slender nanofibers. Because the nanofibers have been internalized, a strong immune system can be activated without adjuvants. When T-cell and B-cell epitope peptides are added to peptides, epitope peptides can function as adjuvants, making them an attractive option for developing treatments for a variety of diseases. Wu, Y. et al. (68) studied Coil29 (QARILEADAEILR-AYARILEAHAEILRAQ), a nanofiber with a long alpha helix in which four peptides form a square in each of its spiral stacks, with the C-terminal end clustered near the nanofiber axis and the N-terminal end extending toward the nanofiber surface (**Figure 3C**). APCs can internalize coil29 nanofibers to activate T cell immunity. At the same time, polypeptide nanoparticles containing CD4+ T cell epitope peptides can stimulate site-specific antibody responses (68). Q11-PA CD8+ epitopes (H2N-SSLENFRAYV-SGSG-QQKFQFQFEQQ-Am) are composed of β-folded supramolecular polypeptide nanoparticles, where Q11 is a β-folded polypeptide, and PA is a restricted epitope of MHC-1. Intranasal administration of PAQ11 increased the duration of action of CD8 T cells (69).

Immune checkpoints are a pathway for T cell activation. PD-L1 is overexpressed in a variety of cancer cells and binds to PD-1 in immune cells to inhibit the immune activity of T cells. One tumor suppression strategy is to reduce the consumption of T cell phenotypes by blocking the interaction between PD1 and PD-L1, restoring normal T cell activity. Based on this strategy, Xiao, W. Y. et al. (70) developed a peptide immune checkpoint blocking strategy (CRICB) by linking the PD-L1 targeting peptide TP (SC (DBCO)FPNWSLRPMNQM) with the assembled peptide AP (MDEKAQKGPAKLVFFAC (Cy)EK (N3)G) via azides (**Figure 3D**). The spontaneous aggregation of TP-AP *in situ* was formed, and the occupying time of PD-L1 was prolonged (70). In addition, Liu, H. et al. used phage libraries to screen out four peptides with high specificity for PD-L1, among which CLP002 and CLP003 can highly block the mutual effect of PD-1 and PD-L1, which was beneficial for restoring the T cells proliferation and preventing apoptosis (13).

Retinoblastoma binding protein (RbAp48, RBBP4 or NURF55) is a tumor suppressor. Phosphorylation of RBAP48-derived peptide S249/T252 mimics the interaction with NF-κB, inhibiting the activation of NF-κB and inhibiting the expression of PD-L1 in an NF-κB-dependent manner to activate the role of T cells in tumors (85).

In addition to innate immune function, HNPs attract T cells and initiate a T cell-dependent immune response (26). Furthermore, HNPs promote Ag-specific IgG and increase IgM Ab levels in serum, stimulate CD4+ T cell proliferation, and trigger the release of IL-5, IFN-γ, IL-6, and IL-10 (71). HNP1-3 can significantly increase the production of specific antigen IgG in mice, while HBD2 can boost the immune response to viruses (71). Studies have shown that binding HBD2 or HBD3 to the N and C ends of spik-like peptides, membrane-like peptides, and cu-shaped peptides can increase vaccine immunogenicity without allergic effects, which allows defensins to bind T cell and B cell epitope peptides to develop SARS-CoV-2 vaccines (86).

### 2.3 Other immunity strategies

Allergen therapy refers to inducing tolerance to the allergen by giving small doses of the allergen extract over a period of time. T cell epitope peptides with allergen immune advantages can reduce the side effects caused by allergens. Polypeptide immunotherapy (PIT) uses short peptides composed of immunodominant T cell epitopes of major sensitizing proteins. These peptides do not activate mast cells and basophils but instead activate T cells. T-p8 (YLVLASLIACS) and T-P10 (SNVASLKGFIT) peptides from Per a 10 can reduce IgE expression, promote T cell proliferation (87), and demonstrate good therapeutic potential in allergic mice (88). Bet v 1 (89) is an allergen found in birch pollen. It lacks IgE reactivity and allergen activity, which can induce the body to produce allergen-reactive IgG and inhibit the combination of IgE and allergen.

Granular hemolysin is produced by cytolytic T lymphocytes (CTL) and NK cells to mediate cell lysis and achieve sterilization. CD8 CTL lyzes macrophages infected with mycobacterium tuberculosis through a granulose-dependent mechanism (90). d-31-50v44w is a peptide derived from granulohemolysin whose helix-ring-helix structure is modified by replacing valine in the 44th amino acid with tryptophan. Experimental results show that d-31-50v44w has better antibacterial action and reduces the cytokine and chemotaxis caused by *Bacillus acnes* (91).

Oncolytic peptides can act directly on tumor cells and release DAMPs such as Calrein, ATP, HMGB1, mtDNA, and formyl peptides after tumor killing. These DAMPs bind to specific antigens on APCs and are then delivered to T cells to activate the body’s immunity. LTX-315 (KKWWKKWDipK-NH2) is a 9-mer cationic peptide derived from the cationic antimicrobial peptide bovine lactoferrin according to structure-activity relationship analysis, which has direct tumor killing function and low activity against normal cells (92, 93). LTX-315 acts immunologically by killing tumor cells, releasing DAMP to activate T cells, and altering the tumor microenvironment (92). At present, LTX-315 has entered phase I clinical trials.

## 3 The application strategies of immunopeptides in ocular diseases

### 3.1 Dry eye disease

The prevalence of dry eye disease (DED) is estimated to be 20%-50% worldwide, mainly caused by reduced tear secretion and excessive tear loss (94, 95). Pathological examination showed that DED corneal function decreased, CD4+T lymphocyte infiltration in the conjunctiva increased, conjunctival epithelial cell apoptosis increased, and conjunctival GC mucin decreased. The pathogenesis of DED is complex and may be the product of multiple factors, which may involve autoimmune diseases, chronic inflammation, infection, contact lenses, body hormone changes, and environmental factors. Studies on the mechanism of DED have found that the role of innate immunity is bidirectional: it aggravates the pathogenesis of DED or improves the symptoms of DED (96).

Th17 helper T cells and IL-17A are the crucial factors of DED. Macrophages act as APCs in DED to facilitate the migration of Th17 helper T cells through blood vessels to the ocular surface (97). Meanwhile, APCs produced cytokines (IL-1β, IL-6, IL-21, IL-23, and TGF-β) to drive the differentiation of Th17 cells (98), and IL-6 promotes T cells to differentiate into Th17 cells. Moreover, Th17 cells migrate to the ocular surface through the CCR6/CCL20 axis, and disruption of CCR6/CCL20 binding has been found to alleviate the degree of DED (99, 100). Therefore, CCR6/CCL20 can be a therapeutic target for DED. Th17 is the dominant cell in DED, which releases IL-17A and IL-22 to participate in the regulation of DED and releases IL-5 and IL-7 to prolong Th17. IL-17A promotes lymphangiogenesis in the corneal epithelium and enhances the anti-IgM and anti-CD40 effects to promote B cell proliferation and thus promote ocular surface inflammation (101, 102). Furthermore, inhibition of IL-17A significantly reduced the expression of MMP3 and MMP9 and improved the corneal epithelium. In addition, the immune checkpoint PD-L1 in corneal epithelial cells are vital in restricting ocular surface inflammation (103, 104). Blockade of PD-L1 increased T-cell infiltration of the corneal significantly and upregulated chemokines and homologous receptors, resulting in better corneal integrity (103). The interaction network between PD-L1 and Th17 is also expected to be a new target for immunotherapy. PD-L1 can directly inhibit innate immune cells’ liveliness, restraining the initiation and differentiation of autoreactive T cells into inflammatory effector Th17 cells. The next attempt may be the immune peptides targeting PD-L1 and T cells reviewed above.

**Figure 4:**
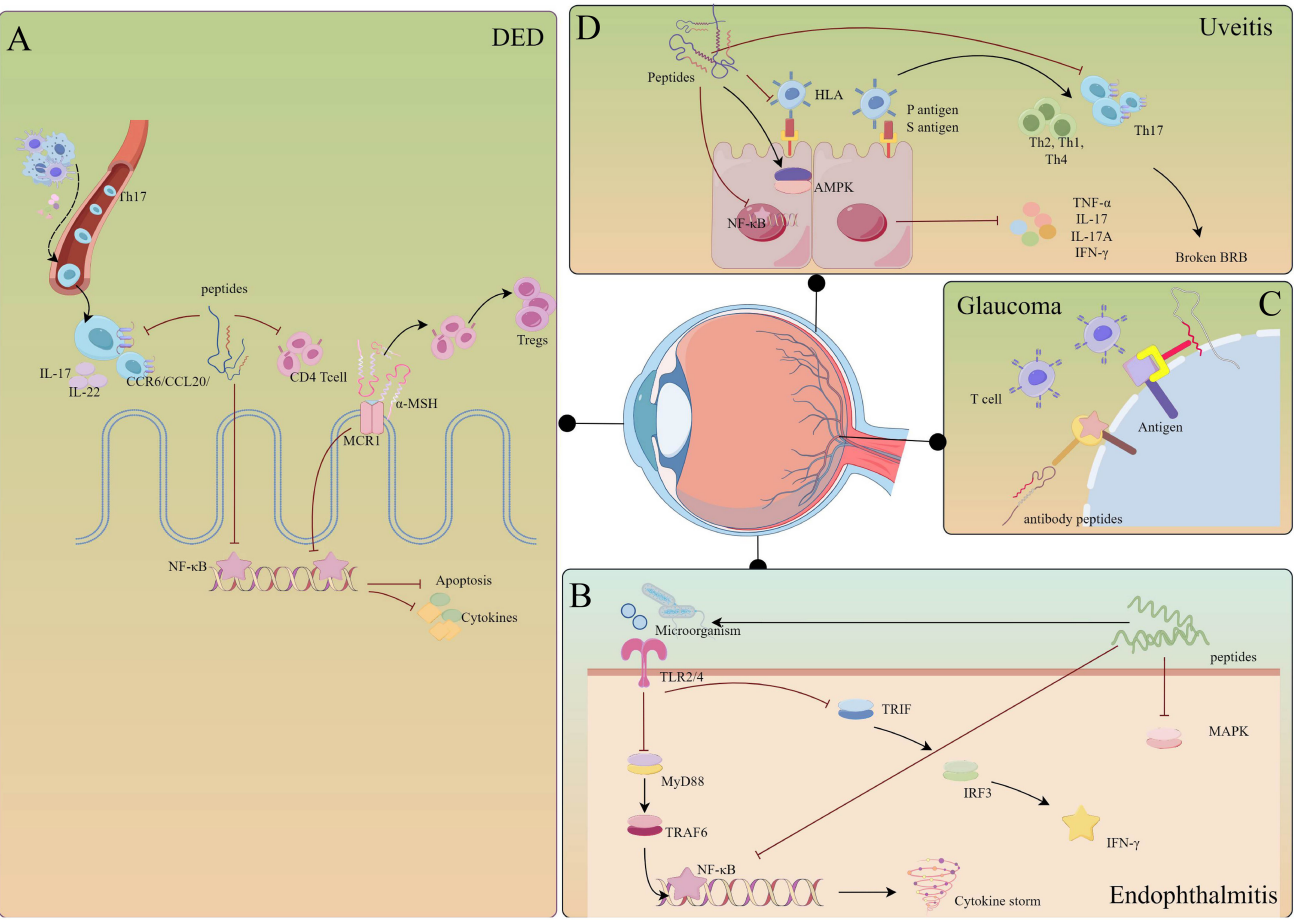
Mechanisms of immunopeptides involved in the regulation of eye diseases. **(A)** DED: immunopeptides inhibit the migration of Th17 cells and the activation of CD4+ T cells. Meanwhile, immunopeptides inhibit the NF-κB pathway, preventing corneal epithelial cells from apoptosis and releasing cytokines. Especially, α-MSH prompts T cells to transfer into Tregs. **(B)** Uveitis: immunopeptides prevent the activation of HLA and NF-κB, activating AMPK to limit autoimmune inflammation. Furthermore, immunopeptides reduce the infiltration of Th17 cells, preventing BRB breaking. **(C)** Glaucoma: immunopeptides act as antibodies that competitively bind to antigens on the cell membrane. **(D)** Endophthalmitis: immunopeptides could combine with LPS, inhibiting the activation of TLRs and down-streaming pathways. The figure was drawn By Figdraw.

Currently, treatment for DED involves supplementing tears and applying local anti-inflammatory agents. However, these treatments only provide temporary relief, making it crucial to create new drugs for DED patients (**Figure 4A**).

Histatin (Hst), a cationic protein rich in histidine, is encoded by the HTN1 and HTN3 genes and is present in saliva and tears. This protein plays a crucial role in the homeostatic regulation of the host defense system, exhibiting antibacterial functions and regulatory effects on epithelial damage and periodontitis (105). At the ocular surface, histatin in tears was negatively correlated with the severity of DED (106). Lee, S. M. et al. found in RAW264.7 that Hst1 impeded the macrophage release of proinflammatory cytokines and MAPK signaling pathways and reduced nitric oxide (NO) release to protect ocular surface tissues (39). In the mouse SDS model, it was found that Hst5 could reduce the loss of conjunctival PAS mucin, diminish SDS-induced infiltration of CD45-positive cells, and lower the rate of conjunctival TUNEL-positive cell apoptosis (107). Whether Hst5 regulates the infiltration of different immune cells in DED requires further investigation.

**Figure 5:**
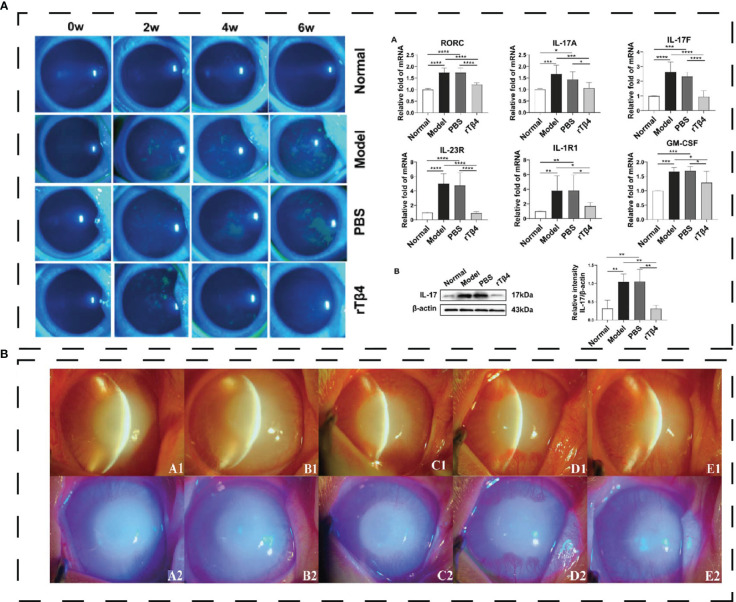
**(A)** Effect of polypeptide on DED. rTβ4 repaired corneal injury and regulated Th17 *in vivo* (111); *P < 0.05, **P < 0.01, ***P < 0.001, ****P < 0.0001. **(B)** the eyes with alkali burn on day 21 (A1-A2: 10 μg/ml GP; B1-B2: 20 μg/ml GP; C1-C2: 40μg/ml GP; D1-D2: rh-EGF; E1-E2: PBS) and diffuse light observation with blue cobalt light (112). Reprinted with permission from journal/author license.

Thymosin β4 (Tβ4) is one of the antimicrobial peptides in exterior ocular defense in thecal and conjunctival epithelial cells. Tβ4 is a G-actin-binding protein consisting of 43 amino acids, accounting for 70% to 80% of the total amount of thymosin in humans. Tβ4 can promote corneal wound healing (108), regulate inflammatory mediators *in vivo*, and reduce the infiltration of polymorphonuclear neutrophils (109, 110). Germinal peptide (GP), one of the active sites of Tβ4, can treat rabbit corneal injuries and inhibit corneal inflammation (**Figure 5B**) (112). Mechanistically, Tβ4 inhibited the NF-κB pathway and the downstream transcription mediated by TNF-α (113). Zhai, Y. et al. designed recombinant human thymosin β4 (rhTβ4) to treat mice DED model induced by benzalaceum chloride (BAC) (114). rhTβ4 down-regulates conjunctival inflammatory cytokine and CD4 T cell expression by hampering NF-κB activation while promoting tissue repair by reducing MMP family protein expression and the apoptosis of conjunctival cells (114). The infiltration of Th17 cells in the ocular surface can aggravate DED, and inhibition of Th17 cells to release cytokines can alleviate the symptoms of DED. In a rabbit model, the Recombinant Tβ4 protein can inhibit the expression of IL-17A and GSM-F in Th17 cells in the lacrimal gland and inhibit STAT3 signal transduction (**Figure 5A**) (111). Thymosin β4 eye drops (RGN-259) are safe and effective in treating severe DED in phase 2 randomized trials (115).

The discovery of corneal nerve immune crosstalk has enriched the study of corneal immunity. As Wu M. et al. reviewed (116), the outermost layer of the cornea is innervated by abundant nerves that control ocular surface processes, such as TRPV1 and PIEZO1 proteins that regulate tear film evaporation, tear osmolality, and tear secretion. Corneal nerves are not only related to the production of tear and blink reflexes to sustain lubrication and the integrity of corneal epithelium but also express neuropeptides such as calcitonin gene-related peptide (CGRP), substance P (SP), and vasoactive peptide to regulate corneal inflammation and fight bacterial infection (116). The mechanism between nerve stimulation and corneal neuropeptides has provided new insights into the immunotherapy of corneal immune diseases.

Vasoactive intestinal polypeptide (VIP) is a kind of neuroendocrine peptide that can be secreted by corneal nerve innervation and released by innervated and activated immune cells in lymphocytes. VIP exerts anti-inflammatory functions through four pathways (117): 1. Inhibit the production of pro-inflammatory cytokines by macrophages and microglia. 2. Up-regulation of the strong anti-inflammatory cytokine IL-10. 3. Inhibition of macrophage activation of T cells. 4. Inhibition of Th1 cell response. VIP can directly act on CD4+ T cells to promote the liberation of Th2 transcription factors and inhibit Th1 transcription factors, and VIP can promote the development of Th2 memory cells (117). In C57BL/6 mice, VIP up-regulates the expression of anti-inflammatory factors, growth factors, and TLRs during corneal infection (118). VIP also generates DCs to regulate inflammation with a tolerogenic phenotype, promotes T cell differentiation to Th2 cells, and reduces the transition to Th1 and Th17, increasing CD4+ and CD8+ Tregs (119). The ability of VIP to modulate corneal inflammation suggests that VIP holds promise as an immune peptide for treating DED.

Melanocortin receptor-targeting peptide has potential translational value as an alternative to non-steroidal drugs in the treatment of DED. The endogenous melanocortin neuropeptide α-melanocyte-stimulating hormone (α-MSH) is secreted by the RPE, ciliary epithelium, and iris and circulates in the anterior chamber and vitreous (120). Structurally, melanocortin peptides share the core sequence HFRW, which is essential for binding to the receptors (121). Melanocortin binding receptor 1 (MC1R) is widely exposed in corneal epithelium, RPE and Müller cells, and has the strongest binding to α-MSH and is involved in inflammation and immunosuppression (120). In EUA mice model, α-MSH/MCR1 could inhibit the secretion of IFN-γ, promote T cells to converse into Tregs, and curtail the ability of effector T cells to activate and proliferate (73). α-MSH/MCR1 acts on macrophages to inactivate NF-κB and thus reduce the expression of pro-inflammatory cytokines and chemokines (73). α-MSH and its derivatives have been investigated for the treatment of DED. PL-8331 targeting MCR1, MCR3, MCR4, and MCR5 can inhibit corneal inflammation and maintain corneal integrity in the dry eye C57BL/6J model (122). In a phase 2, 12-week study, PL-9643 can alleviate the symptoms of moderate to severe DED and reduce corneal and conjunctival damage (123).

### 3.2 Immunological peptides defense pathogens and promote wound healing in Corneal infections

Infection-driven corneal inflammation is the key to effective treatment of keratitis. KAMPs control the infection and inflammation of the corneal simultaneously, which can target TLR and inhibit the activation of NF-κB, IRF3, and proinflammatory factors (124). Moreover, nerves can be affected rapidly during infectious keratitis, and SP shows high relevance in infectious keratitis. Research is definite that SP promotes HSV-1 infiltration and latency in trigeminal neurons (125), and SP can enhance the severity of *P. aeruginosa* keratitis (126). So, inhibitors targeting the SP-binding domain may be a new therapeutic approach.

Tβ4 has the function of regulating corneal inflammation. In *P. aeruginosa* keratitis, ciprofloxacin and adjunctive Tβ4 treatment groups inhibit the release of ROS and lipid peroxidation significantly. Moreover, Tβ4 can reduce the PMN invasion to the cornea and change the pro-inflammatory response of the anti-inflammatory response (127).

**Figure 6:**
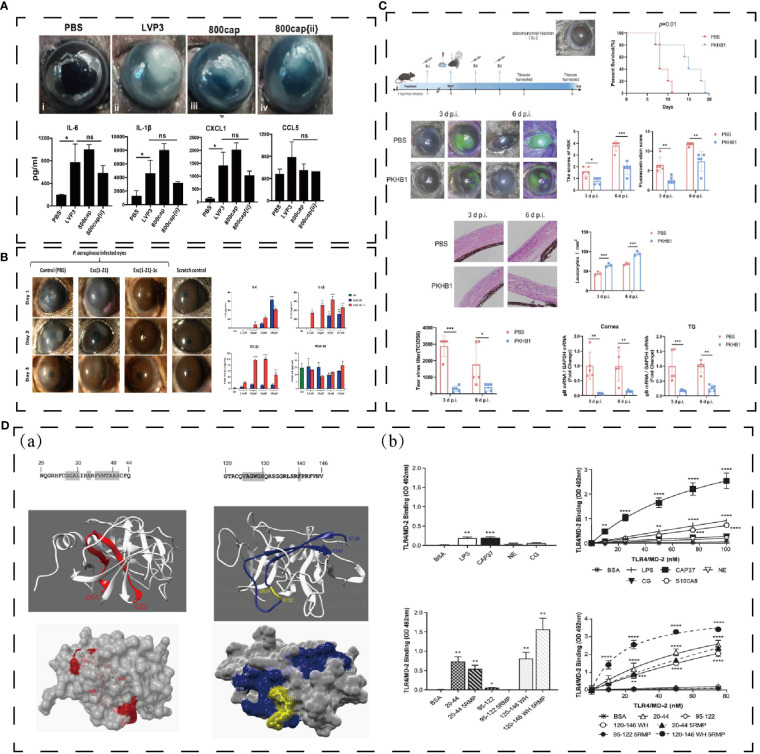
**(A)** Effect of polypeptide on keratitis. 800cap treated corneal infection and down-regulated cytokines and chemokines (129). ***p < 0.0005; ns denotes not significant;. **(B)** Esc (1–21)-1c reduced corneal infiltration and mediated cytokines and growth factor (130). * p < 0.05, ** p < 0.01, *** p < 0.001, and **** p < 0.0001. **(C)** PKHB1 peptide inhibited HSV-1 replication and alleviated HSK lesions (131). *P < 0.05, **P < 0.01, ***P < 0.001. **(D)** Structure of CAP37 and derived peptide, and they directly bind TLR4/MD-2 *in vitro* (132). *P < 0.05, **P < 0.01, ***P < 0.001. Reprinted with permission from journal/author license.

Tetraspanins (CD9, CD81, CD63, Tspan21 and CD151) can be antibacterial targets, and a CD9 EC2-derived synthetic peptide, 800, shows the ability of reducing Staphylococcus aureus adherence to keratinocytes in human skin models (128). Further study defined that CD9-derived peptides were useful to treat mice corneal *P. aeruginosa* infections and promote to release cytokines and chemokine (**Figure 6A**) (129).

Peptide Esc (GIFSKLAGKKIKNLLISGLKG-NH2) is a membrane-active peptide, which is secreted from the Pelophylax lessonae/ridibundus frog. Esc(1-21)can light the bacterial infection of cornea and Esc(1-21)-1c can treat keratitis elicited by Pseudomonas infectious in a mouse model and contribute to healing the corneal wound *in vivo* corneal debridement wound (**Figure 6B**) (130).

PKHB1 (kRFYVVMWKk) is a peptide similar to 4N1K, which combined with CD47 to mediate immune cell and innate immunity (133, 134). In herpes simplex keratitis (HSK) mice model, PKHB1 activates the ICD to release more cytokines (iNOS, CD86, IFN-γ, IL-1β, TNF-α and IL-6) to fight against immune evasion (**Figure 6C**) (131).

CAP37, a cationic antimicrobial peptide, also has the facilitate to corneal protection. Based on its structure and function, H. Anne Pereira et al. has designed CAP37-derived peptide, which inhibits the S100A9/TLR4 pathway in C57/BL6 model (**Figure 6D**) (132). S100A8 and S100A9 are correlated with bacterial keratitis (132). CAP37 might be beneficial for therapeutic use in corneal injuries and infections.

### 3.3 Application prospect of immune peptides in infectious endophthalmitis

Infectious endophthalmitis is a severe inflammation caused by bacterial or fungal infection in the vitreous cavity that may lead to permanent vision loss (135). The global incidence of postoperative acute endophthalmitis is 0.036%-0.360% (136). Acute bacterial endophthalmitis often develops rapidly within 24 hours and is characterized by eye pain, redness, vision loss, destruction of intraocular structures and tissues, and even enucleation (136, 137). Inflammation triggered during endophthalmitis will cause irreversible damage to the retina. Therefore, it is important to target inflammatory factors in treating endophthalmitis (**Figure 4B**).

In bacterial endophthalmitis, innate immunity activates macrophages, glial cells, and Müller cells to regulate inflammation, increasing the infiltration of neutrophils and macrophages to clear invading pathogens. TLRs triggers innate immunity in all retina layers, and regulated the downstream inflammation (138). The classical TLR pathway forms a signaling axis with MyD88, which activates NF-κB and MAPK signaling pathways through ubiquitination of transforming growth factor kinase 1 (TAK1) by the ubiquitin ligase TNF receptor-associated factor 6 (TRAF6), releasing downstream cytokines, such as IL-6, IFN-γ, and TNF-α. IL-1β, MIP-1α, GRO, and GCSF and chemokines CXCL1, CXCL2, and CXCL10 were involved in the regulation of inflammation (137, 139). However, the cascade reaction of inflammatory factors can easily cause irreversible damage to the intraocular tissues, resulting in permanent loss of vision. The non-Myd88-dependent pathway, also known as the TRIF1-dependent pathway, regulates immune response through IFN-I (140). Different microorganisms activate different inflammatory pathways in the eye’s posterior segment, so treatment combining antibacterial and anti-inflammatory functions is the best effect. Gram-positive bacteria activate MyD88-NF-κB signaling mainly through TLR2 in endophthalmitis (141) and G- can activate innate immunity through both the classical MyD88 and non-myd88 pathway (141). Fungal endophthalmitis destroys the BRB, which allows ARPE to act as the initial defense to coordinate innate and adaptive immunity (142). ARPE promotes immune responses by activating PRRs such as CLR and TLR. This process involves the activation of NF-κB and MAPK signaling cascades (143) and the increasing of downstream cytokines and chemokines such as TNF-α, IL-1β, IL-6 and CXCL2 (144). In addition, PMN infiltration is increased in the vitreous cavity of fungal endophthalmitis. PMN production of NADPH oxidase (NOX) and ROS can enhance fungal clearance (145) while it aggravates the damage of the retina (144).

**Figure 7:**
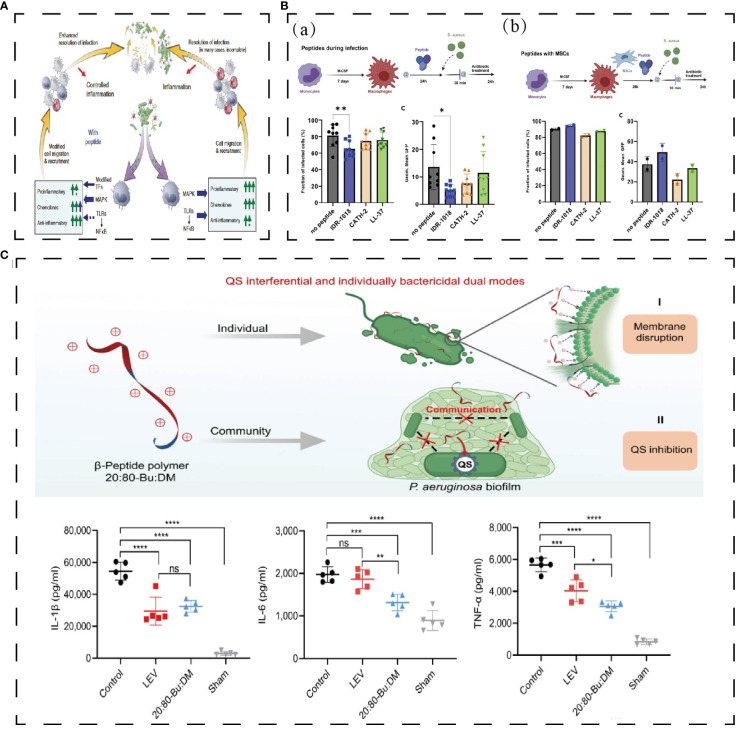
**(A)** Effect of polypeptide on antibacterial and anti-inflammation. Bacteria activates innate immunity, leading to the activation of the TLR-NFkB and MAP kinase pathways (12). **(B)** IDR-1018 reduced the number of bacteria phagocytosed by macrophages and IDR-1018 wouldn’t influence macrophages’ phagocytosis when combined with MGNs (147). **p < 0,01, *p < 0,02. **(C)** 20:80-Bu: DM inhibits biofilm and reduces bacteria virulence by its anti-QS activities and reduces proinflammatory cytokines (148). *P < 0.05, **P < 0.01, ***P < 0.001, ****P < 0.0001. Reprinted with permission from journal/author license.

HDPs have not yet been utilized in the study of infectious endophthalmitis, but their anti-inflammatory and antibacterial properties hold promise for treating the condition. Endogenous HDPs are secreted by epithelial cells and immune cells during tissue infection with bacteria or fungi, improving the outcome of the infection (146). CATH-2, IDR-1018, and LL-37 can kill *S. aureus* directly and activate LPS-mediated macrophage to decrease the expression of TNF-α and IL-10 (**Figure 7B**) (147). IDR1 (KSRIVPAIPVSLL-NH2) is a chemotactic immunoregulatory peptide which is chemotactic to neutrophils, monocytes, and macrophages (146). IDR1 plays an immunomodulatory role and reduces the expression of pro-inflammatory factors through MAPK and other pathways. At the same time, IDR1 shows a good therapeutic effect on MRSA and vancomycin-resistant *Enterococci* (**Figure 7A**) (12). 20: 80-Bu: DM, a membrane-targeted dual-modality HDP, can effectively interfere with the quorum sensing and biofilm formation of *Pseudomonas aeruginosa* and down-regulate the expression of IL-6, IL-1β, and TNF-α in pulmonary infection (**Figure 7C**) (148). The HDPs with both antibacterial and anti-inflammatory functions described above are meaningful attempts to treat infectious endophthalmitis and will not be described in detail here (**Table 1**).

In addition, another strategy to indirectly activate HDPs has been used in antibacterial and anti-inflammatory studies. Wang, J. et al. screened 15 porcine HDP-inducing compounds by high-throughput screening. These inducing compounds (Xanthohumol, isorhapontigenin, deoxyshikonin, calycosin, sorhapontigenin and so on) induced HDPs without causing significant increases in proinflammatory cytokines. It can also increase the body’s antibacterial activity (149). Butyrate, a human gut microbial metabolite, attenuates the proinflammatory effects of mouse bone marrow-derived macrophages (BMDMs) and Müller cells and indirectly regulates the expression of LL-37 (150).

### 3.4 Glaucoma

Glaucoma is a degenerative disease of the retinal ganglion. In addition to high intraocular pressure, antibodies, CD4 T cells, and heat shock protein (HSP) produced by the microbiome may be the pathogenesis of glaucoma (151). One mechanism of glaucoma autoimmunity is (152): High intraocular pressure directly causes optic nerve injury and induces CD4 T cell infiltration. The HSP, produced by the intestinal flora, cross-reacts with the epitope protein of retinal ganglion cells (RGC), serving as the target antigen of T cell response. Therefore, it is of great significance to investigate the mechanisms of neuroimmune protection to protect RGCs from ocular hypertension, CD4 T cells, and HSP. At present, the primary method of glaucoma drug treatment is to reduce intraocular pressure, but there is little treatment for optic nerve degeneration.

There are two immune strategies for treating predominantly optic neurodegenerative glaucoma (**Figure 4C**): 1) transfer of T cells that destroy antigens in the eye and optic nerve; 2) active vaccination with immune antigens without inhibiting T cell activity. Inhibition of T cells will affect the immune regulation of optic nerve injury but will be more susceptible to RGC damage. Therefore, active vaccination with immune antigens and protection of T cells or activity will be a better immune strategy. Peptide-copolymer 1 (Cop-1) (153) is a synthetic peptide that cross-reacts with myelin antigens. It is clinically used as an immunosuppressant in myelin-related autoimmune diseases and can replace myelin antigens to protect the optic nerve from damage. Schori H. et al. demonstrated that immunization with Cop-1 enhanced T cell autoimmune responses and protected retinal ganglion cells from death by establishing a glaucoma mice model of glutamate damage to the optic nerve (154). R16 peptide (ADGSSWEGVGVVPDV), derived from the 1177-1191 sequence of photoreceptor retinoid-binding protein, is an immunodominant antigen present in the eye, which can reduce glutamate-induced optic nerve damage (74). Schmelter C. et al. used liquid chromatography-mass spectrometry to analyze CDR peptides (complementarity-determining regions) that have immunoregulatory effects on optic nerve cells, among which CDR1 (ASGYTFTNYGLSWVR) exhibited neuropreservation activity on RGCS *in vitro*, with increased release of anti-apoptotic and antioxidant protein genes and decreased expression of stress-related HSP (155).

HSP is one of the natural ligands of MHC II, and peptides targeting MHC II may help inhibit its effect. However, HSP70 has also been found to inhibit proinflammatory factors. HSP70 can inhibit NF-κB signaling pathway, which is dependent on inflammatory stimulation, prompting the down-regulation of MCP-1, IL-6, and IL-8 in synoviocytes of arthritis patients after TNF-α stimulation (156, 157). Therefore, the immunotherapy strategy against HSP should be carefully selected in glaucoma.

### 3.5 Non-infectious uveitis

Uveitis is a potentially blinding intraocular inflammatory disease within the eye caused by infection or autoimmunity. It comprises more than 30 diseases, with endophthalmitis as the primary manifestation (158) (reviewed in Jabs, D. A et al. (158)). According to the etiology, it can be divided into infectious uveitis and non-infectious uveitis. Non-infectious uveitis results from an imbalance between regulatory and inflammatory mechanisms in the immune system. The uvea is rich in blood vessels and pigments in structure, and the uvea has a wide area and slow blood flow, so immune-active substances are easy to deposit here. The abnormal structure of uveitis and uveitis antigen components such as S antigen, P antigen, and IRBP can induce the uveitis sensitization reaction (159). Immune active substances with HLA to T helper cells will stimulate T cell to activate, leading to differentiation into Th2, Th1, Th17, and Treg cells (75, 160–162). Th2 cells as control factors of Th1 cells, secretion of IL - 4 and anti-inflammatory factors such as IL - 10, and suppress Th1 tissue damage caused by hyperactivity (161, 163). Th1 cells produce IFN-γ and IL-2, which are subject to regulation by other cytokines, such as IL-17 and IL-10 (164). As mentioned earlier, IL-17 secretion by Th17 cells is important in initiating inflammatory and autoimmune diseases (165).

The production of granzyme B by Th17 can cause the destruction of BRB and the breakdown of the retinal neurovascular unit (NVU), resulting in the destruction of the immune microenvironment and the recruitment of lymphocytes and macrophages to the uvea, thereby aggravating uveitis (166). Decreasing the pathogenicity of Th17 cells could improve autoimmune uveitis (167). However, targeting IL-17A in the treatment of uveitis is ineffective, as IL-17A deficiency does not reduce the pathogenicity of Th17 cells in uveitis but rather increases the expression of GM-CSF and IL-17F (168). Ke Y et al. have demonstrated that dysfunction or dysregulation of CD4+CD25+ Treg cells are essential in the recurrence or progression of autoimmune uveitis in rats, and CD4+CD25+ Treg cells are involved in the alleviation of intraocular inflammation (169).

Chronic, non-infectious uveitis requires long-term suppression of inflammation. Currently, steroids and anti-inflammatory drugs are used to treat uveitis, but the nephrotoxicity and other adverse reactions of drugs are not unsuitable for long-term use. Therefore, immunopeptides, as safe and low-toxic biomaterials with immune regulation, have great potential for treating uveitis (**Figure 4D**).

**Figure 8:**
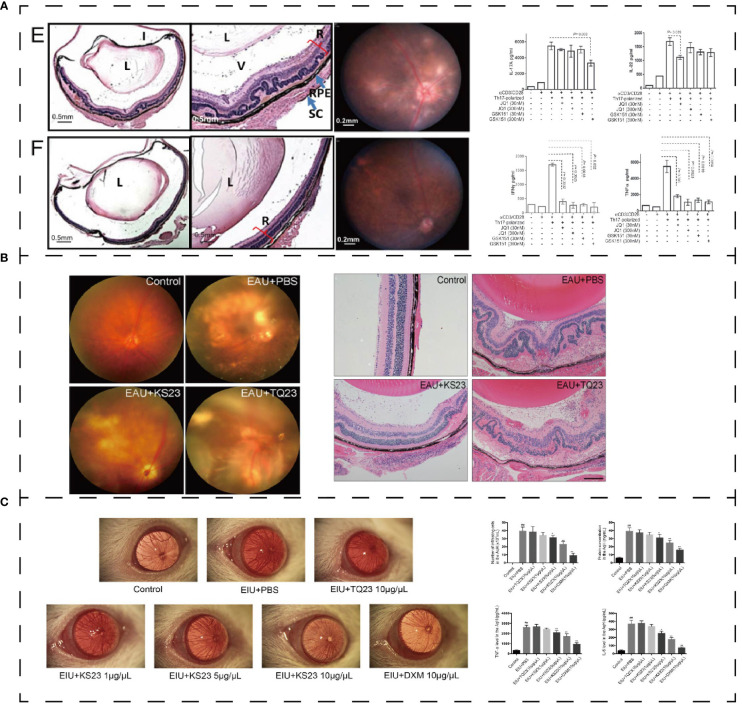
**(A)** Effect of polypeptide on uveitis. BET inhibitor ameliorated inflammation in EUA (E: control group; F: GSK151) and mediated IL-17A, IL-22, IFN-γ, and TNF-α (171). **(B)** KS23 suppresses the development of EAU (75). **(C)** K23 treated EIU and mediated pro-inflammatory cytokines in the aqueous humor (172). *p < 0.01 vs. the control group. **p < 0.05 vs. the LPS group. ##p < 0.01 vs. the LPS group. Reprinted with permission from journal/author license.

Uveitis is thought to be mediated by autoreactive T cells that are specific to retinal proteins. One potential approach for treating inflammatory and autoimmune diseases is to inhibit the differentiation of Th17 cells and their subsequent functions. Targeting the bromodomain and extraterminal (BET) proteins, which bind to acetylated lysine residues in histone and non-histone proteins, shows promise for treating malignant tumors and chronic inflammation (170). In experimental autoimmune uveitis (EAU), two BET inhibitors (GSK151 and JQ1) have been found to alleviate the inflammatory progression and CD4+ T cells. A 5-days treatment of BET inhibitors decreased the expression of IL-17A, IL-22 and retinoic acid-related orphan receptor γt in Th17 cells (**Figure 8A**) (171).

Adiponectin (APN) is a polypeptide composed of 244 amino acids secreted by adipocytes (173). It activates Th1 and Th17 cells under normal conditions but has a negative effect on T cell activation in autoimmune diseases (173). Based on the optimization of APN function, Niu, T. et al. (75) designed an innovative 23-aa immune peptide extracted from the globular C-terminal domain of APN: KS23 (KDKAMLFTYDQYQENNVDQASGS). KS23 can improve the progression of EAU in B10RIII mice. KS23 significantly reduces the release of TNF-α, IFN-γ, IL-17 and IL-23A. And it decreases the proportion of Th1 and Th17 cells in peripheral blood by activating AMPK while inhibiting NF-κB signaling pathway to limit autoimmune inflammation (**Figure 8B**) (75). In the EIU model, KS23 ameliorated inflammation by inhibiting IKKα/β/IκBα/NF-κB pathway and inhibited the secretion of TNF-α and IL-6 in the aqueous humor (**Figure 8C**) (172).

Rho guanine nucleotide exchange factor (GEF-H1) can stimulate RhoA to regulate epithelial and endothelial barrier function and cell migration. Inhibition of GEF-H1 can effectively regulate the inflammation of endothelial cells, and GEF-H1 is up-regulated in the RPE of uveitis patients (174). Based on GEF, Mills C. et al. (175) designed a GEF-H1 peptide inhibitor (TAT-P5) with therapeutic effect on EAU. TAT-P5 inhibited the progression of experimental autoimmune uveitis in mice (175). Therefore, GEF-H1 can be used as a therapeutic target to improve disease outcomes.

The neuropeptide α-MSH is secreted by the RPE, ciliary epithelium, and iris and circulates in the anterior chamber and vitreous. As mentioned above, α-MSH mediates anti-inflammatory and immune processes and regulates the immune process of Treg cells through melanocortin receptors. Therefore, α-MSH receptor agonists have great potential in the treatment of EAU. Ng, T. F et al. used four kinds of melanocortin receptor agonists to treat EAU and found that melanocortin receptor agonists could protect the retina of EAU mice by activating the α-MSH receptor (122). When EAU mice were treated with α-MSH, APCs in the spleen reversed retinal antigen-specific Th17 cells into inducible Treg cells (176).

Since antigens need to bind to HLA molecules and be presented to T cells, HLA peptides are an important link in treating uveitis. Thurau, S. R (76). developed particular therapeutic strategies based on HLA peptides: B27PD (ALNEDLSSWTAADT), derived from HLA-B antigen sequences, is similar to retinal S-antigen peptides (FLGELTSSEVATEV, S-Ag), which induce specific suppressor cells in the gut to release cytokines to inhibit the effector T cells and ocular inflammation. The HLA peptide is low in triggering an autoimmune response as it has a weak uveogenic effect and induces much less mucosal immunity than whole proteins (76). Currently, oral B27PD has been used in clinical trials (177). Analysis of the structure and binding domain of HLA molecules to inhibit T cell activation or antigen binding by immune tissue may be necessary for the treatment of uveitis.

### 3.6 Uveal melanoma

Uveal melanoma (UM) is an aggressive malignant tumor that originates from melanocytes in the uvea, mainly in the choroid, followed by the ciliary body and iris (178). The immune-privileged environment of the eye would be conducive to tumor growth, which allows highly immunogenic tumor cells to survive and grow in the eye. The infiltrating cells of Ums are mainly CD8+ T cells and macrophages. UMs express pigment-associated antigens, like melanoma antigen 1 (MART1), tyrosinase, tyrosinase-associated protein 1 (TRP1) and melanocyte protein PMEL (gp100), which can be recognized by T cells (178). Therefore, antigens expressed by Ums provide new targets for treatment.

Tebentafusp, an immunomodulation that targets gp-100, a specific antigen of UMS, is being tested on January 25, 2022, in adult patients with HLA-A* 01:1-positive unresectable or metastatic uveal melanoma (179). Gp-100 is a transmembrane glycoprotein highly expressed in normal melanocytes and melanoma cells. Tebentafusp is an immune-mobilization monoclonal TCRs anticancer molecule (ImmTACs), a bis-specific gp100 peptides-HLA-directed CD3 T-cell conjugative fusion protein (180). Tebentafusp was designed based on the tumor-associated peptides-human leukocyte antigen complex (pHLA). However, the T-cell receptor TCR: CD3 scFv complex can recognize HLA-presented 8-to 15-amino acid peptides (181). pHLA is a cancer-specific target library, and the TCR is cross-bound in the HLA binding groove. So that the TCR complementarity determining region 3 (CDR3) is centrally located in the antigenic determinant (peptide), and the CDR1/2 loop is mainly located on the HLA helix, enabling the native TCR to detect pHLA in a peptide-dependent manner (182). Tebentafusp, which is linked by a disulfide bond between an alpha and beta chain, is specific for the core sequence of GP-100, YLEPGPVTA, and redirects and activates T cells to lyse target cells (180). *In vitro* experiments demonstrated that Tebentafusp exerted anti-tumor effects through adoptive cellular immune response with the release of IL-2, TNF-α, and IFN-γ (183). In addition, lymphocytes are attracted and activated and dendritic cells are induced to mature, which capture tumor-associated antigens of apoptotic tumors and bind to T cells to lyse more cancer cells (184).

## 4 Prospect

At present, part of the immune peptide has been used in DED, ocular surface, uveitis, and uveal melanoma research. A new generation of polypeptide drug design will combine innovative chemical synthesis methods, a calculation model, and a variety of peptide structures. One challenge in peptide design is the ability of immune peptides to compete for binding to natural targets, which increases the difficulty of binding of immune peptides to target proteins due to the specific affinity of endogenous polypeptide ligands for natural targets. There are two strategies to improve immune efficacy: one is to optimize peptide ligands to recruit different immune cells; the second is to block antigen binding to HLA receptors by HLAs blockers (185). Meanwhile, the limitations of peptides are inescapable. The stability of proteolysis, poor absorption, and transport properties limit the administration of polypeptide therapy. Also, lack of specificity, resulting in poor selectivity and undesired side effects. Recently, peptidomimetics have been developed to display metabolic stability, good bioavailability, high receptor affinity, and selectivity (186).

Add natural amino acids such as lysine, arginine, and hydrophobic amino acid residues can effectively improve the affinity of immune peptides and lower hydrolysis enzyme activity of peptides (187, 188), as specific amino acids can restrict the spatial structure of a polypeptide to achieve its optimal activity (189). Amino acid sequence by peptide database of high flux screening can effectively filter out the immune activity of the peptides. Hancock et al. incorporated non-natural cationic amino acids of different side chain lengths into IDR1018 using a peptide array synthesized by SPOT and screened peptides with unique immunomodulatory activities (52).

Associated with autoimmune disease (DED, Ums, and uveitis) and HLA-present related immune (uveal melanoma) process, the molecular structure of the analysis of HLA proteins determines the practical anchor point in HLA molecules as the combination of the polypeptide targets. Zhou, Q et al. analyzed that the side chains of 263F and 272E in the collagen II (CII)-derived peptide are mainly coupled to aa on HLA-DR1 chains by hydrogen bonds through computer models, while TCR might identify the side chains of 267Q and 270K (190). Removing amino acids recognized by the TCR and maintaining the intact HLA-recognized residues, T cell stimulation can reduce T cell stimulation, and an anti-inflammatory response can occur (190). The platform on which Tebentafusp was developed, ImmTACs, is also the core sequence of the alternative antigen that binds to the HLA receptor, thereby improving T-cell immunity and tumor apoptosis (180–182).

The use of innovative computer algorithms facilitates high-throughput screening of peptides. SuPepMem (https://supepmem.com/) is an open-access repository that can undertake peptide molecular dynamics simulation, thus finding a relationship between peptides and membrane function (191). Another database, PepTherDia (Peptide Therapeutics and Diagnostics: http://peptherdia.herokuapp.com), analyzed the characteristics in detail and collected information about its terminal half-life, plasma protein binding, indications, dosage, method of production, marketing authorization (approved), and year for the first time, as well as the design source of information (14). *Phage display technology* is a high-throughput peptide analysis method that enables unbiased sampling of billions of peptides on phages. Therefore, the structure, function, and biology of peptides can be studied by using this technology. According to the different peptide identification modules, peptide-phage simulation method is used to collect protein-protein networks at the core of the sequence, which are then collected into a database (http://www.prm-db.org) (192). Using the method of high-throughput screening and database optimization to optimize the peptide structure and function will provide new prospects for immune therapy for eye diseases.

## 5 Conclusion

Due to the BRB, the human eye forms an immune-privileged environment, and the activity of immune cells in the eye is in delicate balance. Eye diseases caused by immune problems greatly affect people’s lives and even vision loss. The core sequence of immune peptides to activate immune regulation comes from the combination of natural peptides or amino acids with immune effects, giving immune peptides an advantage in regulating the human body’s immune system. In micro-organisms infectious diseases (infectious keratitis and endophthalmitis), HDPs can kill drug-resistant bacteria and regulate the inflammatory process caused by infection, which is beneficial to the treatment and prognosis of infectious diseases. In adaptive immune processes (DED, UMS, uveitis, glaucoma), the sequence of immune peptides is close to or complementary to endogenous peptides, and immune peptides can act on antibodies or antigens to activate T cell immunity and regulate autoimmune diseases or tumors. In addition, immunopeptides have the characteristics of low molecular weight, easy synthesis, weak toxicity, and low cost. At the same time, there are many databases about peptides and strategies to optimize effective immunity peptides. Therefore, immunopeptides are expected to promote the immune regulation of eye diseases to a new stage of treatment.

## Author contributions

YT: Visualization, Writing – original draft, Writing – review & editing. SQ: Conceptualization, Visualization, Writing – review & editing. ZN: Visualization, Writing – review & editing. HW: Funding acquisition, Project administration, Writing – review & editing.
